# Midichlorians - the biomeme hypothesis: is there a microbial component to religious rituals?

**DOI:** 10.1186/1745-6150-9-14

**Published:** 2014-07-02

**Authors:** Alexander Y Panchin, Alexander I Tuzhikov, Yuri V Panchin

**Affiliations:** 1 Institute for Information Transmission Problems, Moscow, Russian Federation; 2 Department of Ophthalmology, Bascom Palmer Eye Institute, University of Miami, School of Medicine, Miami, FL, USA; 3 A.N. Belozersky Institute Of Physico-Chemical Biology, Moscow State University, Moscow, Russia

**Keywords:** Microbiome, Religion, Brain, Metagenome, Midichlorians, Biomeme, Behavior, Rituals

## Abstract

**Background:**

Cutting edge research of human microbiome diversity has led to the development of the microbiome-gut-brain axis concept, based on the idea that gut microbes may have an impact on the behavior of their human hosts. Many examples of behavior-altering parasites are known to affect members of the animal kingdom. Some prominent examples include *Ophiocordyceps unilateralis* (fungi), *Toxoplasma gondii* (protista), *Wolbachia* (bacteria), *Glyptapanteles sp.* (arthropoda), *Spinochordodes tellinii* (nematomorpha) and *Dicrocoelium dendriticum* (flat worm). These organisms belong to a very diverse set of taxonomic groups suggesting that the phenomena of parasitic host control might be more common in nature than currently established and possibly overlooked in humans.

**Presentation of the hypothesis:**

Some microorganisms would gain an evolutionary advantage by encouraging human hosts to perform certain rituals that favor microbial transmission. We hypothesize that certain aspects of religious behavior observed in the human society could be influenced by microbial host control and that the transmission of some religious rituals could be regarded as the simultaneous transmission of both ideas (memes) and parasitic organisms.

**Testing the hypothesis:**

We predict that next-generation microbiome sequencing of samples obtained from gut or brain tissues of control subjects and subjects with a history of voluntary active participation in certain religious rituals that promote microbial transmission will lead to the discovery of microbes, whose presence has a consistent and positive association with religious behavior. Our hypothesis also predicts a decline of participation in religious rituals in societies with improved sanitation.

**Implications of the hypothesis:**

If proven true, our hypothesis may provide insights on the origin and pervasiveness of certain religious practices and provide an alternative explanation for recently published positive associations between parasite-stress and religiosity. The discovery of novel microorganisms that affect host behavior may improve our understanding of neurobiology and neurochemistry, while the diversity of such organisms may be of interest to evolutionary biologists and religious scholars.

**Reviewers:**

This article was reviewed by Prof. Dan Graur, Dr. Rob Knight and Dr. Eugene Koonin

## Background

“A fama discovered that virtue was a spherical microbe with a lot of feet”

- Julio Cortazar, Cronopios and Famas

The human body contains a plethora of microorganisms, including bacteria that inhabit all of our surfaces: the skin, cornea, guts, mouth and sexual organs [1]. Recent research suggests that gut microbes may not only affect certain metabolic processes [2], but also affect the human nervous system and certain behavioral phenotypes including cognition, mood, sleep, personality, eating behavior, and even a variety of neuropsychiatric diseases [3-5]. The human gut microbiome might play an important role in the development of anxiety [6], depression [6] and Alzheimer disease [7]. This phenomenon was labeled “the microbiome-gut-brain axis” [8]. Furthermore, it has been suggested that the modulation of gut microbiota may be a viable strategy for the treatment of certain mental disorders [9]. Lab experiments on animal models show that treatment with specific strains of bacteria may affect the central nervous system by differentially altering GABA receptor expression in several regions of the brain [10]. It was also shown that mice free of specific pathogens display different behavior patterns than germ free mice [11] and that the transfer of intestinal microbiota may influence target host behavior [12]. One suggested mechanism of interaction between microbes and the hosts nervous system is through the production and utilization of behavior-altering neurochemicals, some of which can be found simultaneously in different domains of life [13].

Many parasites are known to dramatically affect the behavior of their animal (metazoan) hosts. *Ophiocordyceps unilateralis* is a fungus that behaviorally manipulates *Camponotus* ants to remain in conditions that are optimal for the fungal development up to the moment when the host dies [14,15]. The *Wolbachia* bacteria are widespread parasites of arthropods and nematodes and may influence their hosts mating behavior in a way that facilitates the reproduction of the parasite [16,17]. Another fascinating example is the crustacean parasite of crustaceans *Sacculina carcini* that can make infected male crabs (*Carcinus maenas*) appear and behave as if they were female, taking care of *Sacculina* eggs as if they were their own [18,19].

The *rabies virus* is capable of moving along axons from the point of initial infection towards the central nervous system where it causes progressive encephalitis [20]. The symptoms induced by the *rabies virus* in animal hosts include dramatically elevated levels of aggression [21] which lead rabid animals to bite, spreading the infection.

Another example is the *Dicrocoelium dendriticum Trematoda* flat worm larvae that appears to modify the behavior of host ants by having them climb tall blades of grass at night and fixate themselves with their mandibles [22]. This behavior makes ants (and the parasite inside) likely to be consumed by cattle, thus spreading the parasite to their definitive hosts. This host-controlling parasite is capable of infiltrating the ants nervous system, typically the subesophageal ganglion [23]. The parasitic *Nematomorph* hairworm, *Spinochordodes tellinii* affects the behavior of its host *Meconema thalassinum* by making this grasshopper more likely to jump into water, where the adult parasite reproduces and the host usually perishes [24]*.* This process was found to be associated with differential expression of proteins specifically linked to neurotransmitter activities in the host [24].

A remarkable example of host control is observed when the larvae of the parasitic wasp *Glyptapanteles sp.* induce their caterpillar hosts (*Thyrinteina leucocerae*) to behave as bodyguards of the parasitoid pupae after the larvae exits the host [25]. It was also shown that this behavioral modification of the host leads to a two-fold reduction in mortality of the parasitoid pupae with no evident benefits for the caterpillar [25]. The *Barley yellow dwarf virus* affects the feeding behavior of *Rhopalosiphum padi* aphids: infected aphids prefer to feed on uninfected plants, spreading the infection [26]. Recently it was shown that the malaria parasite *Plasmodium falciparum* affects the attraction to nectar sources and sugar uptake of the mosquito *Anopheles gambiae*[27].

Around one third of the human population is estimated to be hosts of the parasitic protista *Toxoplasma gondii*[28], although infection rates vary by country [29]. This parasite can be transmitted by cats, the definitive hosts of *Toxoplasma gondii,* and its cysts can be localized in the brain [30]. Although toxoplasmosis is considered to usually have subclinical effects on humans, it was found to be associated with altered behavior in humans and rodents [31,32]. In particular the presence of *Toxoplasma gondii* was linked to suicidal self-directed violence [33] and increased suicide rates in women [34]. Latent toxoplasmosis was found to be associated with increased risk of traffic accidents [35] and with the perception of pleasantness of cat urine odor in a gender-dependent manner in humans [36]. Some associations between personality traits and *Toxoplasma gondii* infection are also gender-specific. For example, infected men are less intelligent and infected women are more intelligent [37], although a casual relation is yet to be proven. Rodents infected by *Toxoplasma gondii* display more preference to feline odors [38,39] and are more likely to expose themselves in the open and become prey for predators. *Toxoplasma gondii* infection has been proposed as a factor that influences human culture, as its latent prevalence explains a statistically significant portion of the variance in aggregate neuroticism among human populations [37].

We reviewed some of the most distinguished behavior-altering parasites from different domains of life. We believe that a few additional parasites should be mentioned exclusively due to their exotic nature despite the fact that they are not currently known to alter host behavior in ways that facilitate their transmission. Prions are agents composed of misfolded proteins that can cause degenerative neurological disorders such as Creutzfeldt-Jakob disease and kuru (also known as the “laughing sickness”) [40]. Kuru is an infectious disease that was transmitted in the course of ritualistic cannibalism in Papua New Guinea [41]. Also a unique infectious cancer in Tasmanian devils (*Sarcophilus harrisii*) while not known to affect host behavior benefits from the preexisting aggressiveness of the host species that allows it to spread [42]. Another notable example of transmissible cancer is the canine transmissible venereal tumor [43].

The variety of parasites that can affect host behavior suggests that the phenomena of parasitic host control might be more common in nature than currently established and could have been overlooked in humans. This warrants a detailed search for parasitic organisms that affect human behavior. One approach to search for “invisible” microbes that influence behavior is by comparing the microbiomes of control subjects and humans that consistently engage in irrational ritualistic behavioral activities, which contribute to the spreading of parasites and infections.

The modern anthropological view on religion is that it is a cultural meme that replicates through social communication [44]. While the meme itself may influence behavior, religious icons are known to be vectors of infectious diseases [45]. Most major religions have rituals that are likely to promote the transmission of infections. This includes circumcision [46], Christian common communion chalice [46], the Hindu ‘side-roll’ [46] and Islamic ritual ablution [46] as well as the Hajj congregation in Mecca [47]. For example, the latter is specifically associated with outbreaks of meningococcal disease [48].

Consider the Hindu ‘side-roll’. It is a ritual that takes place during the 25 days of the Hindu festival period: devoted men lie on the ground and roll sideways around the Nallur temple in Jaffna for approximately 600 meters, usually with exposed torsos. The frequency of ritual performance is significantly associated with Cutaneous larva migrans, also known as creeping eruption of the skin, that is caused by parasitic worm larvae from domestic canine, bovine and feline hosts [49], although recent sanitary measures have reduced the incidence of this particular pathology [50].

Mortification of the flesh and different forms of self mutilation are present in a variety of religious cultures including many indigenous cultures. Some claim that extreme practices of mortification of the flesh may be used to achieve spiritual experiences. Self inflicted wounds as any wounds provide additional routes and possibilities for infections.

“Holy springs” and “holy water” have been found to contain numerous microorganisms, including strains that are pathogenic to humans [51]. The Ganges River probably tops the list. This river is considered sacred in Hinduism, while its waters are considered both pure and purifying and are used for the preparation of traditional medicines. However an estimated 200 million liters or more of untreated human sewage is discharged each day into the Ganges River, its waters contain multiple pathogens [52] and bathing in this water is associated with the development of multiple diseases, including cholera [53].

Also many religions are centered on sacred relics that are worshiped and frequently kissed by multiple people and thereby can act as vessels for microbial transmission. Crosses, icons, Bible covers are kissed in some denominations of the Christian tradition, the Black Stone (the eastern cornerstone of the Kaaba) is a relic that is kissed by millions of Muslims, kissing of the Wailing Wall is a religious tradition for the Jewish. It is unlikely, but possible that the rejection of condom use, vaccination and use of antibiotics present in some religious cultures, as well as the sacred status of specific domestic animals (possible definitive hosts to the parasites) may also be related to microbial host control. Finally, it has been noted that many parasites eliminate their hosts reproductive potential as they channel all available resources to maximize their own reproductive success [18]. Coincidentally celibacy is commonplace for holy individuals that are most devoted to their faith such as monks or nuns.

Thus it is possible that various religious practices could represent biomemes: manifestations of a symbiosis between informational memes [54] and biological organisms. This concept is somewhat similar to the fictional midichlorians of the Jedi Order from the popular series “Star Wars” [55].

Two particular parts of the human body seem to be most promising for the search of behavior-altering parasites. First of all, the human gut microbiome may be of interest in light of the microbiome-gut-brain axis concept. Another promising area to search for behavior-altering parasites is the human brain. Several organisms that can bypass the mammalian blood–brain barrier and produce a latent infection without obvious symptoms are currently known. In mice with latent toxoplasmosis, *Toxoplasma gondii* cysts can be found in various regions of the brain, especially in the olfactory bulb, the entorhinal, somatosensory, motor and orbital, frontal association and visual cortices, the hippocampus and the amygdala [56]. In humans the brain also appears to be an important site for *Toxoplasma gondii* cyst formation and the parasite is capable of infecting a variety of brain cells, including astrocytes and neurons [57-59].

Taeniasis (infection by the larval stage of a pig tapeworm *Taenia solium*) is characterised by mild or no symptoms (most patients are clear of any symptoms) [60], thus the infection can remain undetected. *Taenia solium* commonly infects the central nervous system, causing neurocysticercosis. Epileptic seizures are the most common presentation of neurocysticercosis (actually, a leading cause of seizures and epilepsy in some areas [60,61]) and generally represent the primary or sole manifestation of the disease, however not all patients with diagnosed neurocysticercosis have seizures [60]. Interestingly, clinical observations support an association between religious experiences during, after, and in between epileptic seizures [62]. Most patients with cerebral schistosomiasis are asymptomatic or have mild and nonspecific symptoms [63]. The cerebral infections by the flat worms *Spirometra mansoni* and *Paragonimus westermani* have nonspecific signs and symptoms such as headaches and seizures [63]. Coincidentally, headache status at baseline was found to be positively associated with frequent religious attendance (but not attendance of other social events) in one study [64], consistent with previous findings [65].

*Herpesviridae*, *Paramyxoviridae*, *Rhabdoviridae*, *Picornaviridae*, *Retroviridae* viral families include strains of neurotropic viruses [66,67]. Of these *herpes simplex*[68] and *varicella zoster virus*[69] are known to cause latent infections of nerve tissues. While no one seems to have published any metagenomic projects specifically devoted to the search of microorganisms in seemingly healthy human brains, a metagenomic analysis using cloning and sequencing of 16s rDNA of brain abscesses revealed a number of previously uncharacterized bacteria [70], however their presence is probably related to the observed clinical conditions. In any case it seems that a seemingly healthy brain or a brain of a human with only mild unspecific symptoms might not be as sterile as commonly assumed.

## Presentation of the hypothesis

Some microorganisms would gain an evolutionary advantage by encouraging human hosts to perform certain rituals that facilitate microbial transmission. We hypothesize that certain aspects of religious behavior observed in human society could be influenced by microbial host control and that the transmission of some religious rituals could be regarded as a simultaneous transmission of both ideas (memes) and organisms. We call this a “biomeme” hypothesis.

Other types of behavior may also be influenced by microorganisms. We focus on the example of religious rituals because the facilitation of microbe spreading through some rituals seems evident, their adaptive role for humans is debatable and there seems to be a positive association between parasite-stress and religiosity [71].

## Testing the hypothesis

Our hypothesis makes several testable predictions.

1. We predict that next-generation microbiome sequencing of samples obtained from gut or brain tissues of control subjects and subjects with a history of voluntary active participation in certain religious rituals that promote microbial transmission will lead to the discovery of microbes, whose presence has a consistent and positive association with religious behavior. We believe that this is the most important prediction and it should be tested in a framework similar to other comparative microbiome studies that have become quite common. This association should be casual, thus complying with the reassessed modern version of Kochs postulates [72].

2. Religious behavior could be positively associated with immune system deficiency. For example, while *Toxoplasma gondii* infections are usually latent, cerebral toxoplasmosis becomes one of the most prevailing opportunistic infections in HIV-infected patients [73]. Immune system deficiency makes humans more susceptible to all kinds of parasites most certainly including those hypothetical ones that could affect human behavior. However, it would probably be difficult to find appropriate control subjects to test this prediction. Participation in religious rituals might be more appealing to those lacking good health or could be influenced by various unaccountable social factors. Not to mention the fact that this is a rare case when control subjects may constitute a minority of the world population. However, differences might also be observed between currently healthy individuals with different susceptibility to infection due to inherited factors. For instance, certain SNP variants in human MHC proteins, cytokines and cytokine receptors, as well as the diversity of T-cell receptors and antibodies might be associated with active and voluntary participation in religious rituals.

3. We expect religious behavior to be negatively associated with certain medical treatments against infections (such as antibiotics). We cannot make definite predictions about which treatments might influence the inclination to perform specific religious rituals as we do not know the precise nature of the hypothetical behavior-altering organisms. However, it might be helpful to follow-up the future religious practices of children that have been subjected to different treatments on similar occasions at baseline [74]. Interestingly, our hypothesis also predicts a decline of religious activity in human societies with improved sanitation.

4. There might be changes in religious activity following dietary changes that are known to affect the human gut microbiome composition [75]. Interestingly, fasting is a widespread religious ritual. Fasting is known to reduce total gut bacteria and affect the gut microbiome composition as shown for hamsters [76], Burmese pythons [77], mice [78] and ground squirrels [79].

5. Candidate microorganisms predicted to influence an individual’s participation in religious rituals should also be found on the surfaces of important religious relics or other items involved in these rituals.

It seems that something like *Toxoplasma gondii* would be a good preliminary candidate for the role of our hypothetical microbe that promotes religious behavior as it is prevalent and widespread (as religious practices are) and its infection is associated with some behavioral traits and it is capable of latently residing in the human brain. Coincidentally, the sacred status of cats, definitive hosts of *Toxoplasma gondii* was part of the ancient Egyptian religious tradition for centuries. To our knowledge, no research on the association between toxoplasmosis or similar infections and religiosity has been performed, thus such an association could have been overlooked.

Interestingly, a number of studies have suggested that *Toxoplasma gondii* infection is an important risk factor for the development of schizophrenia and depression in humans [80,81] and in mice models [82]. *Toxoplasma gondii* infected mice display impaired learning, memory capability as well as more severe depression and stereotypy compared to controls [82]. Meanwhile, religious delusions are commonly found in patients with schizophrenia and by comparison with other patients who have schizophrenia, those patients with religious delusions appear to be more severely ill [83]. Some studies report that religion is important for outpatients with schizophrenia and that their religious involvement is higher than in the general population [84], although unaccounted factors for this association may exist.

## Implications of the hypothesis

We acknowledge the speculative nature of our hypothesis that the participation is some religious rituals may be influenced by microorganisms. It is quite possible that our hypothesis will not withstand rigorous testing. However, if proven true, it offers dramatic consequences for society, science, healthcare and religion. The history of science provides a number of examples when conditions that were not expected to have microbial origin turned out to be exactly of that origin. For example, *Helicobacter pylori* infection and not stress or spicy food (as it was commonly assumed) turned out to be the most common cause of peptic ulcer following the findings of Marshall and Warren [85].

Our hypothesis may provide insights into the origin and pervasiveness of certain religious practices. The discovery of novel microorganisms that affect host behavior may improve our understanding of neurobiology and neurochemistry, while the diversity of such organisms may be of interest to evolutionary biologists, psychologists and religious scholars. From the viewpoint of molecular geneticists it would be interesting to study the evolution of these hypothetical organisms and how their genetic variation relates to the variation of religious rituals.

Ethical questions may arise on whether it is appropriate to expose children to microorganisms that influence religious behavior if such exposure can be avoided. Studies will emerge on whether it is possible to modify ones religious behavior by using specifically designed medical interventions (such as antibiotics) or probiotics.

The possible hazards and epidemiological consequences of microorganisms that alter human behavior may need to be modeled [86]. It may be of importance to understand what happens in microbial communities on the surfaces of important religious relics and casings that travel around the world, such as The Gifts of the Magi (relics associated with the life of the saint prophet of the Christian religion Jesus Christ) and popular sacred sites. Such relics and sites interact with hundreds of thousands of individuals from different geographical backgrounds providing a platform for microbial interchange and microbial sex (conjugation). These factors may play an important role in commensal and pathogenic microbial evolution. For example it was recently shown that the spread of certain antibiotic resistance genes in waterborne bacteria is associated with seasonal pilgrimages to sacred sites at the Upper Ganges River [87].

It can be argued that some religious rituals involve washing and bathing, avoidance of potentially contaminated food sources, restrictions on sexual partners and other practices that may actually reduce the parasitic burden of diseases. These forms of behavior might be entirely cultural or even inherent to humans who apparently have a hardwired and adaptive notion of “contagion” [44], or, in some cases, could be a result of competition between different parasites*.* In the case of washing it should be noted that the use of chlorinated or otherwise disinfected water for bathing is a relevantly modern trend, not all natural water sources are pathogen free (consider the Ganges River as an example) and in some cases washing may be associated with parasitic diseases such as schistosomiasis [88]. Ritual washing might be both a way of sanitation and a way for parasite transmission [46,89], depending on the environmental context.

We would like to clarify that our hypothesis does not explicitly suggest the existence of specific microorganisms for each type of religious ritual (although this is possible), but rather the existence of microorganisms that make humans susceptible to acquiring a wide array of somewhat similar irrational ritualistic behavioral traits that facilitate microbial transmission. These behaviors may be shaped and diversified by culture.

It should be mentioned that there is ongoing debate on whether adherence to a certain religion provides a fitness advantage to humans or human societies. In this context we would like to clarify that our hypothesis is not about religion in general, but mostly about specific religious rituals that do not provide apparent benefits to those who perform them, but facilitate microbial transmission. Some examples of such rituals are mentioned in the introduction.

In agreement with our hypothesis, that certain forms of religious behavior may be influenced by microorganisms, a recent finding suggests that “religiosity, as measured by religious affiliation and religious participation and value, was positively correlated with all measures of parasite-stress, and religiosity was correlated more strongly with the prevalence of nonzoonotic infectious diseases than with zoonotic infectious diseases” [71]. Nonzoonotic infections are those that can transfer from humans to humans. Parasite stress is also associated with homicide rates and child maltreatment [90] and predicts authoritarianism in cross-national and cross-cultural samples [91]. Our hypothesis provides an alternative framework and explanation for these findings.

We believe that next-generation sequencing approaches might help narrow down the search for microbes involved in host control especially with the help of automated taxonomic identification tools [92-94] and that the brain and gut microbiomes are of particular interest in the context of our hypothesis.

## Reviewer’s comments

### Reviewer #1 Prof. Dan Graur, Department of Biology and Biochemistry University of Houston

The main question that we have to answer in dealing with the paper by Panchin et al. is “Is the hypothesis presented in this manuscript a scientific hypothesis?” Answering such a question would require an unambiguous answer to the question “What is a scientific hypothesis?” Unfortunately, many answers (some contradictory) have been provided by scientists and philosophers of science alike. A basic feature of a scientific hypothesis according to many definitions is that it is an “educated guess”. That is, the hypothesis is based on prior knowledge and accepted scientific observations. This seems to be the case as far as the Panchin et al. hypothesis is concerned, as the paper contains numerous analogies based on scientific documentation. Additionally, their hypothesis is novel in that it suggests a biological solution for the occurrence of a social phenomenon. Previous explanations invoked memes or genetic traits that are maintained by natural selection. My favorite is outlined in Robin (1973).

For a hypothesis to be considered a scientific hypothesis sensu Popper, it has to be something that can be refuted through carefully crafted experimentation or observation. A key function in the scientific method is deriving predictions from the hypotheses about the results of future experiments, then performing those experiments to see whether they support the predictions. Have Panchin et al. clearly outlined testable predictions? I don’t think their predictions are very clear in this respect. Expecting a “difference” between the religious and the atheists in terms of microbiome is not a very strong prediction. However, that is the best to be expected in the absence of preliminary data.

Can the hypothesis be tested, and that these tests can be replicated? I am not so sure. Usually, a hypothesis should be phrased in the form of an “if, then” statement. The hypothesis presented by Panchin et al. is too crude to be tested by multiple scientists to ensure the integrity and veracity of the evidence.

Finally, we should ask ourselves “What are the chances that the hypothesis will be rejected”. While I doubt that the hypothesis is correct, the predictions derived from the Panchin et al. hypothesis are too open ended for it to be rejected easily.

Are there obvious flaws to the hypothesis? I see two such flows:

The first concerns the clumping of all religions into a single category. Does the hypothesis envisions the same pathogens as etiological agents of Buddhism and Reform Judaism?

The second flaw of the hypothesis concerns a difference between the known behavior-altering parasites described in the paper and the hypothetical religion-inducing parasites required by the hypothesis. For the behavior-altering parasites, we know that the fitness of the carriers is lower in comparison to parasite free non-carriers. In the case of the hypothetical religion-inducing parasites, the situation may be reversed, in the sense that the hypothetical carriers seem to have a higher biological fitness than the non-carriers.

The paper is however thought provoking and amusing and I thank the authors for think outrageous thoughts.

Robin ED. 1973. The evolutionary advantages of being stupid. Perspect. Biol. Med. 16:369–380.

**Author’s response:** We are grateful to Professor Graur for his review and we would like to respond to several of the concerns raised.

1. “Can the hypothesis be tested, and that these tests can be replicated? I am not so sure. Usually, a hypothesis should be phrased in the form of an “if, then” statement. The hypothesis presented by Panchin et al. is too crude to be tested by multiple scientists to ensure the integrity and veracity of the evidence”.

We agree that initially the formulation of our hypothesis and its predictions were a bit too vague. Partially this is because we don’t know which particular types of religious rituals may be influenced by microbes and what is the nature of these microbes. In our revised manuscript we tried to clarify our predictions as much as we can:

“We predict that next-generation microbiome sequencing of samples obtained from gut or brain tissues of control subjects and subjects with a history of voluntary active participation in certain religious rituals that promote microbial transmission will lead to the discovery of microbes, whose presence has a consistent and positive association with religious behavior. We believe that this is the most important prediction and it should be tested in a framework similar to other comparative microbiome studies that have become quite common. This association should be casual, thus complying with the reassessed modern version of Kochs postulates [72]”.

2. “The first concerns the clumping of all religions into a single category. Does the hypothesis envisions the same pathogens as etiological agents of Buddhism and Reform Judaism? “

We thank Professor Graur for this question. We decided to clarify our position in the revised manuscript:

“We would like to clarify that our hypothesis does not explicitly suggest the existence of specific microorganisms for each type of religious ritual (although this is possible), but rather the existence of microorganisms that make humans susceptible to acquiring a wide array of somewhat similar irrational ritualistic behavioral traits that facilitate microbial transmission. These behaviors may be shaped and diversified by culture”.

3. “The second flaw of the hypothesis concerns a difference between the known behavior-altering parasites described in the paper and the hypothetical religion-inducing parasites required by the hypothesis. For the behavior-altering parasites, we know that the fitness of the carriers is lower in comparison to parasite free non-carriers. In the case of the hypothetical religion-inducing parasites, the situation may be reversed, in the sense that the hypothetical carriers seem to have a higher biological fitness than the non-carriers”.

Consider the Hindu side-roll, the bathing in the Ganges River or the act of kissing holy relics. While the adherence to the whole religious systems centered on these rituals might prove advantageous or not, it seems that “throwing away” these rituals would only benefit religious individuals. Also not all religiously affiliated people follow the religious rituals prescribed by their religion or attend religious events. We have added the following text to our manuscript to clarify this issue:

“It should be mentioned that there is ongoing debate on whether adherence to a certain religion provides a fitness advantage to humans or human societies. In this context we would like to clarify that our hypothesis is not about religion in general, but mostly about specific religious rituals that do not provide apparent benefits to those who perform them, but facilitate microbial transmission. Some examples of such rituals are mentioned in the introduction”.

### Reviewer #2 Dr. Rob Knight, University of Colorado, Boulder

This intriguing hypothesis draws on the now well-established principle that parasites and microbes can affect host behavior to suggest that some religious rituals that have the effect of increasing pathogens transmission might in fact be under the control of the pathogens that are thus transmitted, conferring a selective advantage.

The hypothesis is intriguing, and to my knowledge novel.

There are some concerns that should be addressed:

– No actual data are presented in favor of the hypothesis; this would of course strengthen the manuscript considerably.

– We know that gut bacteria (e.g. in Vijay-Kumar et al. 2010 Science, and numerous subsequent studies that should also be cited) can affect behavior, so it is not necessary that microbial differences would be found in brain tissue itself as proposed by the authors.

– Many religious rituals involve washing, avoidance of potentially contaminated food sources, restrictions on sexual partners, etc. Is the proposal that some parasites are better at manipulating host behavior than others, such that particular religious traditions are associated with the parasites they benefit and would there be some competition between those parasites and the parasites that are disadvantaged by the rituals? More thought is needed here.

– It would be interesting to isolate sanitation from other variables that are often correlated with it, but this might be difficult. The authors should consider modeling frameworks that allow conclusions to be drawn from highly confounded and multivariate datasets.

– The taxa identified in the brain transcriptome data are common low biomass/lab contaminants. Substantial additional work, including qPCR, would be needed to confirm these taxa in brain tissue in a convincing way. In general, it is very hard to get correct identifications from shotgun metagenomic sequencing of highly host-contaminated samples, and special procedures analogous to those used for forensic and ancient DNA studies need to be employed to avoid contamination. The alpha proteobacteria are an entire bacterial phylum, and the generalizations about the properties of the very different organisms listed are not helpful and this section should be removed. In general, this analysis is very weak and should either be omitted, leaving this a pure hypothesis paper, or done correctly with careful positive and negative controls. The methods e.g. for taxonomy assignment are also not well described, and in general doing this correctly will take a lot of additional work beyond what is in the present version of the manuscript.

Additionally, some additional connections might be useful to draw:

– Toxoplasma infections have been connected to schizophrenia, which is connected to religiosity, so data confirming/denying the Toxoplasma-religion link might already be available

– Fasting has also been observed to change the microbiome in burmese pythons, mice, and hibernating ground squirrels (and possibly other taxa), this should be mentioned and cited.

– Different components of religious rituals might be affected by different taxa: how correlated are the different components of religious practice from a comparative religion standpoint? e.g. do people who kiss idols also tend to avoid multiple sexual partners?

**Author’s response:** We are grateful to Dr. Knight for his review and we would like to respond to several of the concerns raised.

1. “No actual data are presented in favor of the hypothesis; this would of course strengthen the manuscript considerably.”

The testing of our hypothesis requires large-scale comparative microbiome analysis. What we can do is provide circumstantial evidence. In the original article the following some evidence was provided. “Recent finding suggests that “religiosity, as measured by religious affiliation and religious participation and value, was positively correlated with all measures of parasite-stress, and religiosity was correlated more strongly with the prevalence of nonzoonotic infectious diseases than with zoonotic infectious diseases” [71].

Following up the comment Dr. Knight kindly provided, we have added the possible link between toxoplasmosis, schizophrenia and religious delusions (details will be provided below).

Other than that our hypothesis is based on the widespread phenomena of microbial host control (including the control of humans via the microbiome-gut-brain axis), the facilitation of microbial spread during some religious rituals and a lack of satisfactory explanations for the irrationality of certain types of religious rituals. We agree that this isn’t much, but after all we are proposing a hypothesis that is yet to be tested.

2. “We know that gut bacteria (e.g. in Vijay-Kumar et al. 2010 Science, and numerous subsequent studies that should also be cited) can affect behavior, so it is not necessary that microbial differences would be found in brain tissue itself as proposed by the authors”.

We agree with this comment. We did not make in clear enough in the original manuscript that the gut microbiota is of similar interest; although we did intend this line of thought and we did have a detailed paragraph about the microbiome-gut-brain axis. In the current versions of the manuscript the mentioned and other articles are properly cited. We removed the emphasis on the “human brain microbiome” in our article entirely. After all the gut microbiome might be as important in the context of our hypothesis.

3. “Many religious rituals involve washing, avoidance of potentially contaminated food sources, restrictions on sexual partners, etc. Is the proposal that some parasites are better at manipulating host behavior than others, such that particular religious traditions are associated with the parasites they benefit and would there be some competition between those parasites and the parasites that are disadvantaged by the rituals? More thought is needed here”.

We are very grateful for this idea. We added the following text to the implications section of our article:

“It can be argued that some religious rituals involve washing and bathing, avoidance of potentially contaminated food sources, restrictions on sexual partners and other practices that may actually reduce the parasitic burden of diseases. These forms of behavior might be entirely cultural or even inherent to humans who apparently have a hardwired and adaptive notion of “contagion” [44], or, in some cases, could be a result of competition between different parasites*.* In the case of washing it should be noted that the use of chlorinated or otherwise disinfected water for bathing is a relevantly modern trend, not all natural water sources are pathogen free (consider the Ganges River as an example) and in some cases washing may be associated with parasitic diseases such as schistosomiasis [88]. Ritual washing might be both a way of sanitation and a way for parasite transmission [46,89], depending on the environmental context”.

4. “It would be interesting to isolate sanitation from other variables that are often correlated with it, but this might be difficult. The authors should consider modeling frameworks that allow conclusions to be drawn from highly confounded and multivariate datasets”.

While we believe that this is important, we think that the isolation of different variables depends on the exact procedures involved in a specific experiment, which will depend to a great extent on the population samples properties, sample size and other factors. We don’t think we can give one quick solution to this problem within this article.

5. “In general, this analysis is very weak and should either be omitted, leaving this a pure hypothesis paper, or done correctly with careful positive and negative controls”.

We agree with this criticism of our initial article: we were not able to provide convincing evidence that those microbial sequences from the human brain are not contamination and a much more detailed analysis that falls outside the scope of this article is warranted. This flaw was also mentioned by Dr. Koonin in his review. As suggested by Dr. Rob Knight, we have entirely omitted this analysis from our manuscript. Instead we provided several additional examples of known organisms that can latently survive inside the human brain from existing literature.

6. “Toxoplasma infections have been connected to schizophrenia, which is connected to religiosity, so data confirming/denying the Toxoplasma-religion link might already be available”.

We thank Dr. Knight for this very interesting idea that turns out to be at least partially correct. We have added the following text to our article:

“Interestingly, a number of studies have suggested that *Toxoplasma gondii* infection is an important risk factor for the development of schizophrenia and depression in humans [80,81] and in mice models [82]. *Toxoplasma gondii* infected mice display impaired learning, memory capability as well as more severe depression and stereotypy compared to controls [82]. Meanwhile, religious delusions are commonly found in patients with schizophrenia and by comparison with other patients who have schizophrenia, those patients with religious delusions appear to be more severely ill [83]. Some studies report that religion is important for outpatients with schizophrenia and that their religious involvement is higher than in the general population [84], although unaccounted factors for this association may exist”.

7. “Fasting has also been observed to change the microbiome in burmese pythons, mice, and hibernating ground squirrels (and possibly other taxa), this should be mentioned and cited”.

We are grateful for this comment and we have appropriately cited all of these articles in the current version of the manuscript.

8. “Different components of religious rituals might be affected by different taxa: how correlated are the different components of religious practice from a comparative religion standpoint? e.g. do people who kiss idols also tend to avoid multiple sexual partners?”

This is an interesting question. Unfortunately we were unable to find a satisfactory answer to it in the scientific literature. We believe that this is an interesting topic that warrants further investigation.

### Reviewer #3. Dr. Eugene Koonin, National Center for Biotechnology Information (NCBI, NLM, NIH, United States of America)

The authors of this manuscript present a striking hypothesis, that the interactions between the human host and the microbiome make a substantial contribution to religious behavior. The idea is certainly interesting and much of the article makes for engaging reading. Nevertheless, the present manuscript includes gaps in logic and major weaknesses that compel me to conclude that at the present stage, the idea is sheer, unfounded speculation not a legitimate hypothesis.

I see two fundamental problems with the current narrative. First, although it goes beyond doubt that many parasite can and do alter the behavior of the host organism, the authors do not show a shred of evidence of a specific association between the human microbiome and religion. Surely, religious rituals can and do lead to spread of infections, and so do any other social interactions. Thus, following the author’s logic, one could speculate microbiome-drive evolution of animal sociality in general not religion in particular especially given that non-human animals do not appear to have religion but certainly suffer from parasites and epizootics. However, even that, more logically consistent and less outrageous hypothesis would be the worst kind of adaptationist just so story. There is any number of evolutionary factors behind the evolution of sociality, with the benefits for parasite being only a side effect. The second, even more damning problem is the very notion of “brain microbiome”. I am concerned that all the microbial sequences detected by the authors in brain samples are contaminations. In general, apart from obvious pathologies, brain is a sterile area. The authors are well aware of these weaknesses and of the existence of the blood–brain barrier, and yet, choose to present these findings as some kind of support for their hypothesis.

From my point of view, publication of the current or cosmetically modified version of this manuscript would benefit neither the authors nor the journal. On the bright side, the authors write in a lively, engaging style, especially in the background section. The effects of the microbiome (primarily, the all-important gut microbiome not the imaginary microbiome of the brain) on animal (including human) behavior are certainly of major interest, My suggestion is that this manuscript is thoroughly reworked into a review of this subject. Then, perhaps, it might be appropriate to mention, if only in passing, that the microbiome could affect even such distinctly human activities as religious behavior.

**Author’s response:** We are grateful to Dr. Koonin for his review. We would like to reply to the critical statements of his review.

1. “The authors do not show a shred of evidence of a specific association between the human microbiome and religion”.

What we are proposing is a hypothesis. According to Biology Direct: Hypothesis should present an untested original hypothesis backed up solely by a survey of previously published results rather than any new evidence. Hypothesis should not be reviews and should not contain new data.

Also, it seems that there has been a misunderstanding. We are not discussing religion in general, but we are talking about specific behavior, participation in religious rituals (as stated in the title of our article). We hope that we made this clearer in the revised version of our article.

The testing of our hypothesis requires large-scale comparative microbiome analysis. What is within our reach given the available data is to provide circumstantial evidence that somewhat supports our hypothesis. In the original article some evidence was provided. “Recent finding suggests that “religiosity, as measured by religious affiliation and religious participation and value, was positively correlated with all measures of parasite-stress, and religiosity was correlated more strongly with the prevalence of nonzoonotic infectious diseases than with zoonotic infectious diseases” [71]”.

Following up the comments made by Dr. Knight, we added the following paragraph that also supports our principal idea.

“Interestingly, a number of studies have suggested that *Toxoplasma gondii* infection is an important risk factor for the development of schizophrenia and depression in humans [80,81] and in mice models [82]. *Toxoplasma gondii* infected mice display impaired learning, memory capability as well as more severe depression and stereotypy compared to controls [82]. Meanwhile, religious delusions are commonly found in patients with schizophrenia and by comparison with other patients who have schizophrenia, those patients with religious delusions appear to be more severely ill [83]. Some studies report that religion is important for outpatients with schizophrenia and that their religious involvement is higher than in the general population [84], although unaccounted factors for this association may exist”.

Finally, there is evidence that certain latent infections of the brain produce nonspecific symptoms such as headaches and seizures. Headaches are associated with religious attendance while seizures are associated with certain religious experiences. References and details are provided further below.

Other than that our hypothesis is based on the widespread phenomena of microbial host control (including the control of humans via the microbiome-gut-brain axis), the facilitation of microbial spread during some religious rituals and a lack of satisfactory explanations as to why people perform these rituals. We agree that at the moment there isn’t much evidence to support our hypothesis, but after all it is a novel idea that is yet to be tested.

2. “Surely, religious rituals can and do lead to spread of infections, and so do any other social interactions”.

We agree that this is true; however, most social interactions have apparent benefits for those who attempt to engage in them. The religious rituals discussed in our manuscript and other irrational interactions are “special” because they provide no apparent benefits for anyone but the hypothetical microbes.

3. “Thus, following the author’s logic, one could speculate microbiome-drive evolution of animal sociality in general not religion in particular especially given that non-human animals do not appear to have religion but certainly suffer from parasites and epizootics”.

We emphasize that we are talking about “religious behavior”, “irrational behavior”, “behavior that has no rational explanation”. Animals do engage in this kind of behavior, in particular when they are infected with parasites. Consider one of the examples of animal behavior given in our manuscript: the ant climbing a blade of grass every night. This is no different from “religious behavior” in the context of our hypothesis.

4. “I am concerned that all the microbial sequences detected by the authors in brain samples are contaminations”.

This concern has also been raised by Dr. Knight and we agree that we did not provide sufficient evidence to exclude contaminations in the brain sequences that we analyzed. Following Dr. Knights suggestion we decided to omit this part of our article. After all it is not crucial for our hypothesis if the hypothetical microorganisms that influence religious behavior turn out to live in the brain or in the gut. We have entirely removed the emphasis from the “brain microbiome” concept in our manuscript.

5. “In general, apart from obvious pathologies, brain is a sterile area. The authors are well aware of these weaknesses and of the existence of the blood–brain barrier, and yet, choose to present these findings as some kind of support for their hypothesis.”

We respectfully disagree with the notion that the seemingly healthy human brain is necessarily a sterile area. We have decided to address this issue in our manuscript by providing additional examples of organisms that can reside in a seemingly healthy human brain and making this statement more clear.

“Two particular parts of the human body seem to be most promising for the search of behavior-altering parasites. First of all, the human gut microbiome may be of interest in light of the microbiome-gut-brain axis concept. Another promising area to search for behavior-altering parasites is the human brain. Several organisms that can bypass the mammalian blood–brain barrier and produce a latent infection without obvious symptoms are currently known. In mice with latent toxoplasmosis, *Toxoplasma gondii* cysts can be found in various regions of the brain, especially in the olfactory bulb, the entorhinal, somatosensory, motor and orbital, frontal association and visual cortices, the hippocampus and the amygdala [56]. In humans the brain also appears to be an important site for *Toxoplasma gondii* cyst formation and the parasite is capable of infecting a variety of brain cells, including astrocytes and neurons [57-59].

Taeniasis (infection by the larval stage of a pig tapeworm *Taenia solium*) is characterised by mild or no symptoms (most patients are clear of any symptoms) [60], thus the infection can remain undetected. *Taenia solium* commonly infects the central nervous system, causing neurocysticercosis. Epileptic seizures are the most common presentation of neurocysticercosis (actually, a leading cause of seizures and epilepsy in some areas [60,61]) and generally represent the primary or sole manifestation of the disease, however not all patients with diagnosed neurocysticercosis have seizures [60]. Interestingly, clinical observations support an association between religious experiences during, after, and in between epileptic seizures [62]. Most patients with cerebral schistosomiasis are asymptomatic or have mild and nonspecific symptoms [63]. The cerebral infections by the flat worms *Spirometra mansoni* and *Paragonimus westermani* have nonspecific signs and symptoms such as headaches and seizures [63]. Coincidentally, headache status at baseline was found to be positively associated with frequent religious attendance (but not attendance of other social events) in one study [64], consistent with previous findings [65].

*Herpesviridae*, *Paramyxoviridae*, *Rhabdoviridae*, *Picornaviridae*, *Retroviridae* viral families include strains of neurotropic viruses [66,67]. Of these *herpes simplex*[68] and *varicella zoster virus*[69] are known to cause latent infections of nerve tissues. While no one seems to have published any metagenomic projects specifically devoted to the search of microorganisms in seemingly healthy human brains, a metagenomic analysis using cloning and sequencing of 16s rDNA of brain abscesses revealed a number of previously uncharacterized bacteria [70], however their presence is probably related to the observed clinical conditions. In any case it seems that a seemingly healthy brain or a brain of a human with only mild unspecific symptoms might not be as sterile as commonly assumed”.

6. “The effects of the microbiome (primarily, the all-important gut microbiome not the imaginary microbiome of the brain) on animal (including human) behavior are certainly of major interest, My suggestion is that this manuscript is thoroughly reworked into a review of this subject”.

We would like to emphasize that the idea that microbes can affect the behavior of their hosts is not new and well established, with multiple examples provided in our manuscript including the effects of microbes on human behavior. We merely hypothesized that this well known notion could be extended to explain the existence of some religious rituals that promote microbial transmission.

In our article we have cited several reviews about the gut microbiome and how it affects animal and human behavior and there is not much novelty that we can add to the existing literature on this topic. As we mentioned, we do not hold to the “brain microbiome” concept exclusively. The main novel idea provided in our paper is the hypothesis that religious behavior may be promoted by microbial host control. The idea that these microbes may reside in the brain is secondary.

We also agree with Dr. Koonin that our hypothesis is outrageous and may be incorrect, however we believe that it’s still an interesting one and worth considering. We would also like to notice that our hypothesis isn’t inherently much more outrageous than published ideas that certain behavioral phenotypes including but not limited to cognition, mood, sleep, personality, eating behavior, and even a variety of neuropsychiatric diseases [3-5], as well as suicidal self-directed violence [33] and suicide rates [34], can be affected by microbes. What makes our hypothesis perceived as more outrageous is that religion is indeed a taboo subject in human society.

We are grateful for the compliment on the writing style of our manuscript.

### Response from Dr. Koonin

In the revised version of this manuscript, the authors have removed the dubious data on brain-associated microbes and generally de-emphasized the “brain microbiome”. Certainly, this is a step in the right direction that eliminates some of the most pressing concerns with the original version. I still find the proposed specific link between the microbiome and religious behaviors to be tenuous at best. Nevertheless, the authors are correct in that the origin and survival of such behaviors calls for explanation that might need to go beyond memes.

## Competing interests

The authors declare that they have no competing interests.

## Authors’ contributions

AP conceived the hypothesis and drafted the manuscript. AT made critical contributions to the discussion of the hypothesis and its conception and participated in the drafting of the manuscript. YP also made critical contributions to the discussion of the hypothesis and its conception and participated in the drafting of the manuscript. All authors read and approved the final manuscript.
